# A New Lunar Lineament Extraction Method Based on Improved UNet++ and YOLOv5

**DOI:** 10.3390/s24072256

**Published:** 2024-04-01

**Authors:** Pengcheng Yan, Jiarui Liang, Xiaolin Tian, Yikui Zhai

**Affiliations:** 1 Faculty of Innovation Engineering, Macau University of Science and Technology, Avenida Wai Long, Taipa, Macau, China; 3220004952@student.must.edu.mo (P.Y.); 1809853xii30001@student.must.edu.mo (J.L.); 2 The State Key Laboratory of Lunar and Planetary Sciences, Macau University of Science and Technology, Avenida Wai Long, Taipa, Macau, China; 3 The Department of Intelligent Manufacturing, Wuyi University, Jiangmen 529020, China

**Keywords:** lunar lineament, CCD data, instance segmentation, UNet++ network, YOLOv5 network, polygon-match strategy

## Abstract

Lineament is a unique geological structure. The study of Lunar lineament structure has great significance on understanding its history and evolution of Lunar surface. However, the existing geographic feature extraction methods are not suitable for the extraction of Lunar lineament structure. In this paper, a new lineament extraction method is proposed based on improved-UNet++ and YOLOv5. Firstly, new lineament dataset is created containing lineaments structure based on CCD data from LROC. At same time the residual blocks are replaced with the VGG blocks in the down sample part of the UNet++ with adding the attention block between each layer. Secondly, the improved-UNet++ and YOLO networks are trained to execute the object detection and semantic segmentation of lineament structure respectively. Finally, a polygon-match strategy is proposed to combine the results of object detection and semantic segmentation. The experiment result indicate that this new method has relatively better and more stable performance compared with current mainstream networks and the original UNet++ network in the instance segmentation of lineament structure. Additionally, the polygon-match strategy is able to perform preciser edge detail in the instance segmentation of lineament structure result.

## 1. Introduction

The word lineaments was originally created in the early 20th century, referring to the significant tectonic units in the crust with the formation of minierals, active faults, underground water controls, earthquakes and geomorphology on the earth [1]. As the development of deep space exploration, the academia also found that lineament is one of the common geological structures on the surface of terrestrial planets and their satellites in solar system. Although it has imaging similarity with the lineaments on the earth, the formation mechanism of lineaments on the surface of terrestrial planets and their satellites in solar system is different from that on the earth. Most often, this term refers to linear features on the surfaces of other planets. Lineament structures are usually considered to be closely associated with volcanic activity on the surfaces of terrestrial planets or their satellites. Based on its imaging features and cause mechanism, the lineaments structure contains following main types: ridges, rill, faults, vaills, catena and fractures. Ridges is a complex lineament structure that extend longer and have lower altitudes on the lunar plain. The widely accepted view is that wrinkle ridges are formed by volcanic erosion and tectonic action [2]. Rill and fault are different manifestations under the same causative mechanism [3,4]. These types of lineament structure unusually are associated with lava activity on the Lunar surface [5,6]. Catena is a chain structure consists of regular arrangement and combination of pits in certain interval. It’s usually in a nearly straight line shape. The causes and mechanisms of catena are diverse including surface ring collapsing [7], magma activity under the moon’s surface [8], and meteorite impact [9], they are classified based on the consistent in imaging.

Lunar surface structures are of great significance for understanding and reconstructing the evolution of the lunar geological structure, and the morphology and distribution characteristics of linear structures such as lunar ridges and creeks are closely related to the dynamical tectonic movements in the Moon. The limited number of samples and the difficulty of field surveys make remote sensing the most important means of planetary science research in this area [10]. On the other hand, within the academic community, attention has mainly been focused on the construction of a classification system for linear structures and the study of the causes of different classes of linear structures based on their dynamical mechanisms [11]. Based on the current situation, we designed a new lineament extraction method based on improved-UNet++ and YOLOv5, which aims to improve the detection accuracy and efficiency of lineaments structures on the Lunar surface based on present samples. The main contributions of this paper are as follows:

- A new Lunar lineaments dataset, which is one of the few datasets of its kind, is created. This dataset contains lineament structures based on Charge-Coupled Device (CCD) data from the Lunar Reconnaissance Orbiter Camera (LROC). With a total of 1000 manually collected Lunar lineaments samples, this dataset provides a unique and valuable resource for lunar research. The scarcity of such datasets is due to the complexity and time-consuming nature of the data collection and processing.
- A new lineament extraction method based on improved-UNet++ and YOLOv5 is proposed. It is able to extract lineament structure with relatively better and more stable performance compared with current mainstream networks and the original UNet++ network. The experiment results show that our method increase the accuracy from 0.64 to 0.67, precision from 0.432 to 0.679, maAP@50 from 0.58 to 0.60 and maAP@50:95 from 0.34 to 0.38, IoU from 0.58 to 0.69, mean pixel accuracy from 0.92 to 0.94.
- A polygon-match strategy is proposed to extract lineament structure with perciser edge detail. It is able to perform preciser edge detail in the instance segmentation of lineament structure result.

The rest of this paper is organized as follows. Section 2 introdues the related work. Section 3 describes the proposed method. Section 4 presents the experimental results. Section 5 concludes the paper.

## 2. Related Work

The early studies of lineament structure classification and identification on the earth show that due to the complexity of atmospheric and surface conditions it usually relies on multiple sources of data including Advanced Spaceborne Thermal Emission and Reflection Radiometer (ASTER), Shuttle Radar Topography Mission (SRTM), Digital Elevation Model (DEM), Synthetic-aperture radar (SAR), combining with variety of technic including manual semi-automated and automated algorithms [12]. As a result, single digital image processing might not be competent to produce accurate detection result. The situation is less complicated on the Lunar surface. The Lunar surface is lack of atmosphere and water, it is easier for lunar exploration satellite to collect clear DEM and CCD data [13,14]. The existing lineament structure extraction methods on Lunar surface are mainly based on manual interpretation [5,6,15] and semi-automated algorithms. The former method is usually time-consuming and laborious. It relies heavily on experience and judgment. Often this experience requires a very deep understanding of geology and planetary dynamics. In most situation, this identification is difficult to popularize. The latter category of algorithms is usually based on the characteristics of lineament structure, such as the linear shape, the difference in brightness and the difference in texture. The existing algorithms are mainly based on the above characteristics to extract lineament structure. In early One of the ridge extraction algorithms is based on combination of series digital image processing methods [16]. Due to the specificity of functional design, these semi-automatic algorithms usually perform poorly on some images with complex geological information. This type of lineament extraction method is usually restricted by its specialization ability. It usually has relatively worse performance when dealing with multiple types of landform in same region. Little research have been done to improve the performance of Lunar lineament extraction algorithms. Our previous work focus on classification of lineament structure [17], we did not go further to extract the lineament structure.

The deep learning based method has been widely used in the field of image processing feild [18,19,20]. Especially the YOLO and UNet series networks, it has achieved great success in the field of image classification, object detection and instance segmentation. It has been proved that convolutional neural networks have satisfied result on crater detection based on DEM and CCD remote sensing data. For example, the FasterRCNN [21] has already been applied in object detection with higher efficiency compared with previous work, MSA-YOLO [22] is desgined to object detection in DIOR dataset, RSI-YOLOv5 [23] is applied in remote sensing dataset such as DOTA and NWPU-VHR with outperform results, and a Graph Neural Network (GNN) [24] is desgined to execute the semantic segmentation in satellite image data. It can be concluded that deep learning method has great potential in remote sensing data processing. However, the deep learning methods are mainly applied in crater detection. Very limited attention is attracted to lineament structure identification. Previous work shows that it already made some progress on earth lineament structure [12,25]. As the development of deep space exploration, the Lunar surface exploration satellite has collected relatively considerable amount of high-resolution CCD data. The existing application is able to classify and detect lineament structure from Mars and Moon [17,26]. However, present deep-learning based methods are mainly focusing on crater detection [27,28,29], very few research are related with extraction of lineament structure on the Lunar surface based on deep learning networks due to the lack of such datasets.

In this paper, we subtly navigate through this research gap by proposing an efficient and accurate lineament extraction method based on YOLOv5 and improved-UNet++. This method, specifically designed for the task of lineament structure extraction, leverages the power of YOLOv5’s object detection capabilities and the improved UNet++’s superior performance in semantic segmentation tasks. To support our approach, we have developed a lineament dataset, consisting of high-resolution Lunar surface images with annotated lineament structures. This dataset serves as a valuable resource for training and validating our model. Our experimental results show promising performance of our method, making it a practical tool for processing large amounts of Lunar surface data. These results subtly hint at the untapped potential of deep learning for this complex task, opening up new possibilities for future research in Lunar surface exploration.

## 3. Dataset and Methodology

### 3.1. Dataset Construction

**Figure 1:**
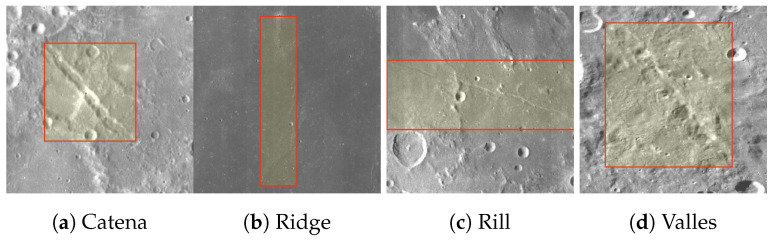
Images of Lineament Structure on the Lunar Surface used in this experiment are captured by Lunar Reconnaissance Orbiter Camera (LROC) [30] at a resolution of 100 m/pix. These images are porvided by NASA/JPL-Caltech. Images above are the typical lineament structure on the Lunar surface. The lineament structure ridge, rills, catena and vallis are the main types of lineament structure on the Lunar surface.

In this experiment, we choose the lineament structure ridge, rills, catena and valles as the content of the dataset. These four types of lineament structure occupied the majority of the known lineament structure on the Lunar surface shown below (Figure 1).

**Figure 2:**
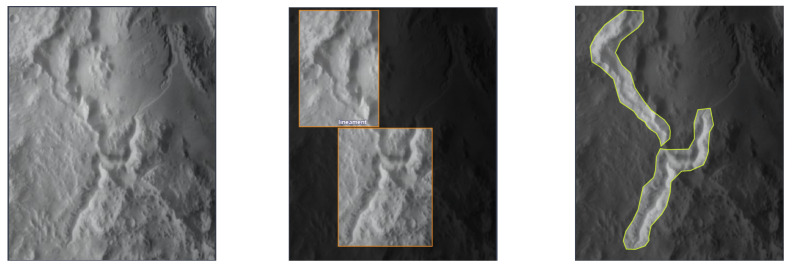
The Visualized Results of the Dataset in this Experiment. The images above visualize the object detection and semantic segmentation results of the lineament structure from the bounding box and binarized mask respectively. The former is used to train the YOLOv5 network and the latter is used to train the improved-UNet++ network.

As shown in Figure 2, in this experiment, we manually labeled the edge of the mentioned lineament structures based on the Lunar Orbital Data Explorer [31]. The manual results are converted into normalized polygon coordinate points through our pre-process program. Those points can be easily converted into object detection labels and binarized mask for semantic segmentation. 1000 lineament samples in total are collected containing all types of mentioned lineament structure for the experiment dataset. Based on the 8:1:1 ratio for training, validating and testing datasets, 800 samples for training set, 100 samples for validation set, 100 samples for test set respectively. The training set and validation set are used to train the improved-UNet++ and YOLOv5 network. The test set is used to evaluate the performance of the proposed method and other instance segmentation algorithms.

### 3.2. Diagram of Algorithm

In this section, we will introduce the proposed algorithm in detail. In the first part, a general view will be given to the proposed algorithm. It will show the lineament extraction algorithm based on instance segmentation contributed by improved-UNet++, YOLOv5, and polygon-match strategy. The following parts will introduce the dataset construction, related network architectures and polygon-match strategy algorithm in detail.

**Figure 3:**
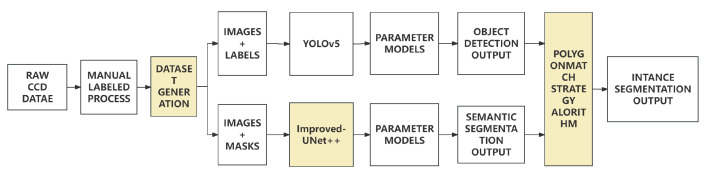
Diagram of Algorithm The marked sections can be regarded as our contribution in this experiment. The contribution can be concluded as following three parts: New lineament dataset construction, UNet++ improvement, and polygon-match strategy algorithm desgin.

The diagram of algorithm is shown in Figure 3. The proposed method mainly consists of three parts: dataset construction network architecture and polygon-match strategy algorithm. The dataset construction part is mainly responsible for the construction of lineament dataset suitable for object detection and semantic segmentation. It consists 2 primary parts: manual labeled process enables to generate the contour of lineaments in json form, dataset pre-process is able to convert previous result into text file form for object detection and binarized mask for semantic segmentation. The network architecture part is mainly responsible for the training of improved-UNet++ and YOLOv5 network. The ’polygon-match’ strategy algorithm part is mainly responsible for the combination of the results of object detection and semantic segmentation. This part is allowed to load trained models from previous part. The final lineament extraction result is generated based on the combination of the results of object detection and semantic segmentation with the correction of polygon-match strategy algorithm.

### 3.3. Network Architecture

#### 3.3.1. YOLOv5 Network Architecture

**Figure 4:**
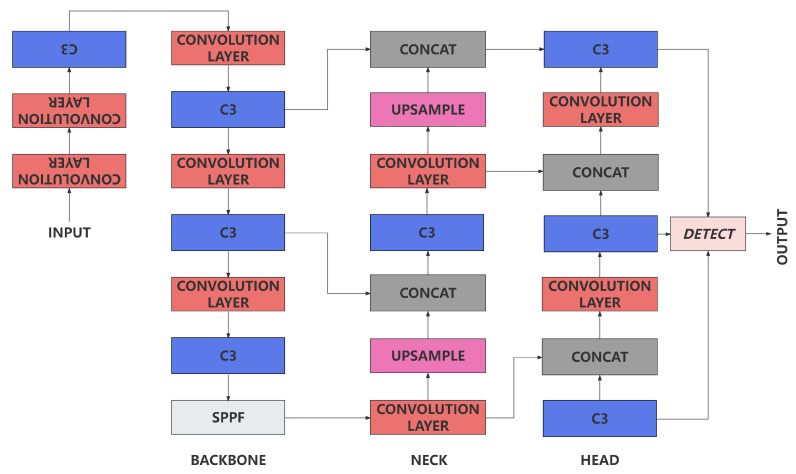
Architecture of YOLOv5.

**Figure 5:**
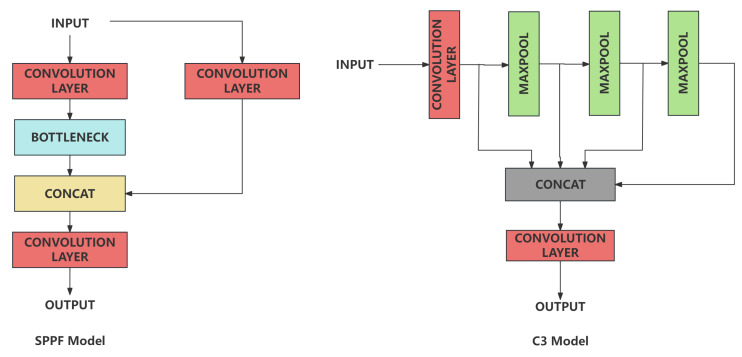
Architecture of C3 and SPPF.

YOLOv5 [32] network is one of the most popular target detection algorithm. Its speed and accuracy outperform its seniors and peers based on its architecture. YOLOv5 consists of three parts: backbone, neck and head. The backbone is usually a convolutional neural network, responsible for extraction of image features. The neck is usually a combination of convolutional layers and upsample layers, responsible for the combination of features extracted by the backbone. The head is usually a combination of convolutional layers and detection layers responsible for the detection of targets. Concat module is used to combine the features of different scales in neck and head, which allowed YOLOv5 to stitch together feature maps at different scales to be spliced. It allows the model to detect targets at different scales simultaneously. The YOLOv5 network architecture is shown in Figure 4. The YOLOv5 network is able to extract features of different scales and combine them to achieve better detection results. It introduces SPPF and C3 into the network architecture innovatively. The SPPF shown in Figure 5 is able to extract features of different scales. The C3 shown in Figure 5 is able to improve the stability and generalization ability of the model. Furthermore, the detect layer is applied as the output layer, it is able to normalize sigmoid output and convert it into bounding boxes location. Also, it engaged Non-Maximum Suppression(NMS), which allowed YOLOv5 to eliminate overlapping bounding boxes. Based on its net engaged modules, the YOLOv5 network is able to achieve better detection results with fewer parameters and faster speed compared with YOLOv4 [33] and YOLOv3 [34].

#### 3.3.2. Improved-UNet++ and UNet++ Network Architecture

**Figure 6:**
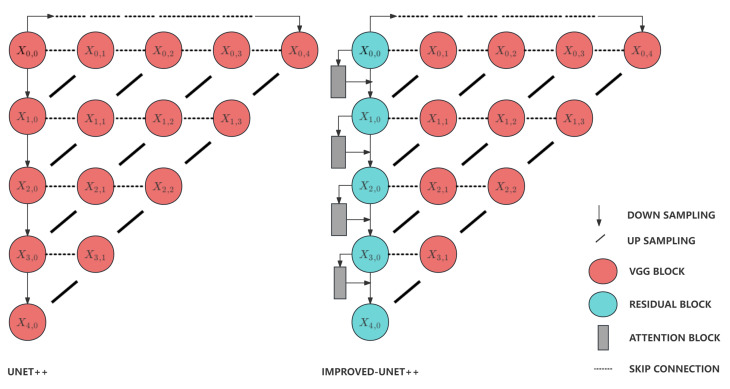
Architecture of UNet++ and Improved UNet++. UNet++ is able to drop the layers, when the model already learns enough features. This type of feature allows the network to have much higher efficiency due to the reduction of the number of parameters from the dropped layers [35].

**Figure 7:**
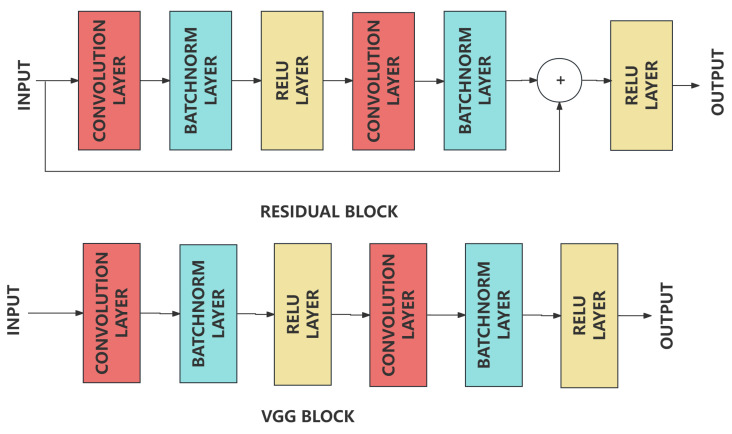
Architecture of VGG Block and Residual Block. The VGG module usually consists of several 3×3 convolutional layers and a 2×2 max-pooling layer, with the step size and padding of all convolutional layers set to 1, making the outputs of the convolutional layers have the same height and width as the inputs. The residual module, on the other hand, consists of two or more convolutional layers and a cross-layer connection, which connects the inputs directly to the layers behind them, thus forming a “short circuit”.

The UNet++ [35] network is one of the most popular semantic segmentation algorithm applied in medical imaging field. In this experiment, we tried to improve the performance of UNet++ by replace the residual blocks with VGG blocks and adding attention blocks in downsampling layers. This type of layer is usually considered as the encoder responsible for lowering the calculation complexity and extracting the distinctive features. The upsampling layers are usually considered as the decoder responsible for the combination of features extracted by the encoder and the recovery of the original image size. Compared with the VGG block, it can be found that a ’shortcut’ path is added in the residual block, which is the key to improve the stability of the network [36]. It has great performance on avoiding the gradient disappearance problem caused by the increase of network depth based on its structure shown in Figure 6 and Figure 7. It follows principles shown below, where $x_{l}$ is the input of the *l*th layer, *F* is the residual block, $w_{F}$ is the weight of the residual block, *L* is the total number of layers in the network, ε is the loss. This type of linear connection between layers guarantees that the loss can be always none-zero value.

(1) $$\begin{array}{cc} x_{l+1} & =F\left(x_{l},w_{F}\right)+x_{l} \end{array}$$

(2) $$\begin{array}{cc} x_{L} & =x_{l}+\sum_{i=l}^{L-1}F\left(x_{i},w_{F}\right) \end{array}$$

(3) $$\begin{array}{cc} \frac{𝜕\varepsilon }{𝜕x_{l}} & =\frac{𝜕\varepsilon }{𝜕x_{L}}\left(1+\frac{𝜕}{𝜕x_{l}}\sum_{i=l}^{L-1}F\left(x_{i},w_{i}\right)\right) \end{array}$$

**Figure 8:**
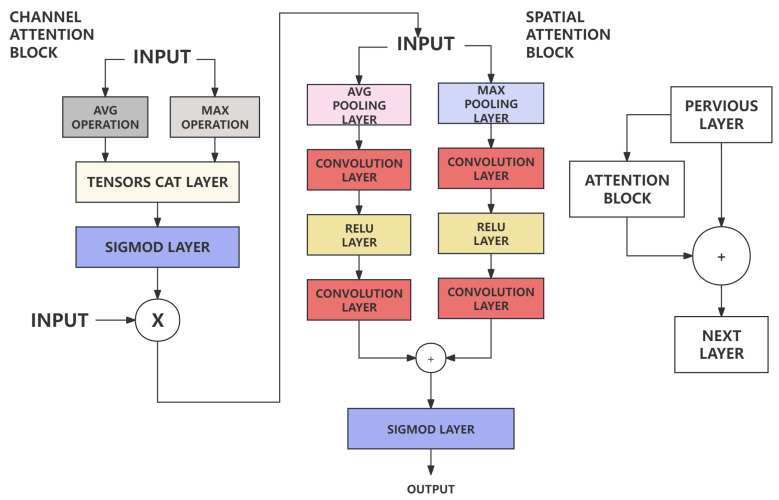
Architecture of Attention Block. Attention block is a combination of channel and spatial attention. It is able to improve the network’s ability to extract features. Due to its felxibility and lightweight, it is widely used in convlolutional layers with very less cost increasing the feature extraction ability at same time [37].

Furthermore, an attention block CBAM(Convolutional block attention module) [37] is added between each layer in the downsampling part of the network, based on its lightweight and versatile features. The attention block is usually considered as a module to improve the network’s ability to extract features. This type of attention model is a combination of channel and spatial. It is implemented based on the following process shown in Figure 8. The output result from previous convolutional layer will first pass through a channel attention module to calculate the weighted result, and then pass through a spatial attention module, and finally the weighted result will be calculated. This type of attention model block is designed based on mathematic principles below. 7×7 is the size of convolutional core in spatial attention block. It shows that 7×7 has better performance compared with general 3×3 core [37].

(4) $$\begin{array}{cc} M_{channelblock}\left(F\right) & =\sigma (MLP(AvgPool(F))+MLP(MaxPool(F))) \end{array}$$

(5) $$\begin{array}{cc} \; & =\sigma (W_{1}\left(W_{0}\left(F_{avg}^{c}\right)\right)+W_{1}\left(W_{0}\left(F_{max}^{c}\right)\right)) \end{array}$$

(6) $$\begin{array}{cc} M_{spatialblock}\left(F\right) & =\sigma (f^{7x7}\left(\left[AvgPool\left(F\right);MaxPool\left(F\right)\right]\right)) \end{array}$$

(7) $$\begin{array}{cc} \; & =\sigma (f^{7x7}\left(F_{avg}^{s};F_{max}^{s}\right)) \end{array}$$

### 3.4. Polygon-Match Strategy Algorithm

**Figure 9:**
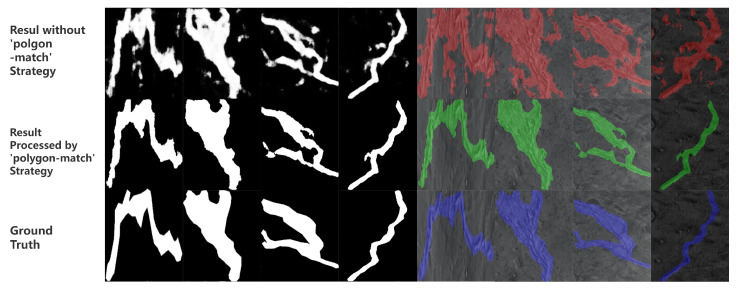
Result of Polygon Match Strategy Algorithm. The binarized mask on the left is the result without polygon-match strategy, result processed by polygon-match strategy and the ground truth. The right part are the visualized results of the binarized masks on the left respectively.

The polygon-match strategy algorithm is mainly responsible for matching the semantic segmentation result with object detection result. The semantic segmentation developed by UNet series networks is based on classification of each pixel with relatively weak understanding of the overall situation. This character usually lead to some random noise on the binarized mask output shown Figure 9 below. From the binarized mask, it can be seen that the binarized mask is not able to accurately outline the edge of the lineament structure, shown in the visualized result. Faced with this situation, we designed the polygon-match strategy algorithm to select the most proper semantic segmentation result based on the object detection result (bounding boxes) from pre-trained YOLOv5 network. This strategy aims to convert the binarized mask result into a list of polygons. For each image, the polygon-match contains following steps:

The polygon-match is designed based on the excellent performance of YOLOv5 in object detection. As shown in Algorithm 1, it is able to detect the area containing lineament structure with high possibility and accuracy. At same time, it is also able to guarantee that each bounding box contains only one lineament structure unit in most situation. As a result, the polygon-match strategy algorithm is able to perform preciser edge detail in the instance segmentation of lineament structure result.
**Algorithm 1** Polygon-match Algorithm1:**while** there are bounding boxes **do**2:   **for** each polygon in the list **do**3:       calculate the area of the polygon4:       **for** each bounding box **do**5:          calculate the IoU of the polygon and the bounding box6:          **if** the polygon is completely contained in the bounding box **then**7:              record the maximum area of the polygon in the bounding box8:          **end if**9:          find the polygon with the largest IoU to the bounding box10:          record the polygon with larger area between the largest polygon contained in the box and the polygon with the largest IoU11:       **end for**12:   **end for**13:   append the selected polygon into a list14:**end while**15:return the bounding box list and the selected polygon

## 4. Experiment Results

### 4.1. Experiment Environment Configuration

This experiment realized based on following hardware and software settings:

- GPU: A4000 x4, RTX3080 x1, RTX3080Ti x1
- CPU: Intel(R) Xeon(R) Gold 6254 CPU @ 3.10 GHz x1,
  Intel(R) Core 10-10900k CPU @ 3.70GHz x2
- Memory: 16 GB DDR4 3200 MHz x4, 32 GB DDR4 3200 MHz x2
- PyTorch: 2.0.1
- CUDA: 12.3
- Batch Size: 16 (for YOLO networks), 4 (for UNet++ networks)
- Epoch: 300
- Learning Rate: 0.001
- Optimizer: SGD (for YOLO networks), Adam (for UNet++ networks)

### 4.2. Evaluation Metrics

In this experiment, we use the following metrics to evaluate the performance of the proposed method and other related algorithms objectively:

- FLOPS: (Floating Point Operations Per Second): Usually, this metric is used to measure the complexity of a model and its time consumption. It is calculated based on the number of floating-point operations in the network and the time consumed by the network.
- IoU (Intersection over Union): IoU is usually used to measure the accuracy of the model. It is calculated based on the intersection area and the union area of the prediction and the ground truth.
- Recall: The ratio of the number of correctly predicted positive samples to the total number of positive samples in the dataset. It is usually used to measure the ability of the model to detect positive samples.
- Precision: The ratio of the number of correctly predicted positive samples to the total number of positive samples predicted by the model. It is usually used to measure the ability of the model to detect positive samples.
- mAP50: The average precision of the model when the IoU is greater than 0.5. mAP is the average of the average precision (AP) of all categories. For each category, AP is the average of precision at different recall levels. It is usually used to measure the accuracy of the model.
- mAP@50:95: The average precision of the model when the IoU is greater than 0.5 and less than 0.95. It is usually used to measure the accuracy of the model.

### 4.3. Experiment Results

In this experiment, in order to ensure the accuracy of the training model and the true generalization ability, we randomly separated the train dataset validation dataset and test dataset with the ration 8:1:1 for 5 times. The average results of 5 times are used for evaluation metrics calculation. Furthermore, in order to verify the effectiveness of our improvement for our UNet++,we desgined an ablation experiment. We trained the original UNet++, orgininal UNet++ with CBAM, original UNet++ with replacing the residual blocks in downsampling layers and our improved-UNet++ with the same data set partitioning and training strategy. The experiment results are shown in Table 1. It can be seen that the improved-UNet++ has relatively better and more stable performance compared with the original UNet++ network in the instance segmentation of lineament structure. At same time, the original UNet++ with CBAM has relatively worse performance compared with the original UNet++ network. It shows that the CBAM is not suitable for the instance segmentation of lineament structure. However, the combination of combination of CBAM and replacing the residual blocks in downsampling layers is able to improve the performance of the original UNet++ network.

Additionally, the polygon-match strategy is able to perform preciser edge detail in the instance segmentation of lineament structure result. The result of the polygon-match strategy algorithm is shown in Figure 9. It is able to improve the IoU from 0.37 to 0.6601, based on our experiment result.

**Figure 10:**
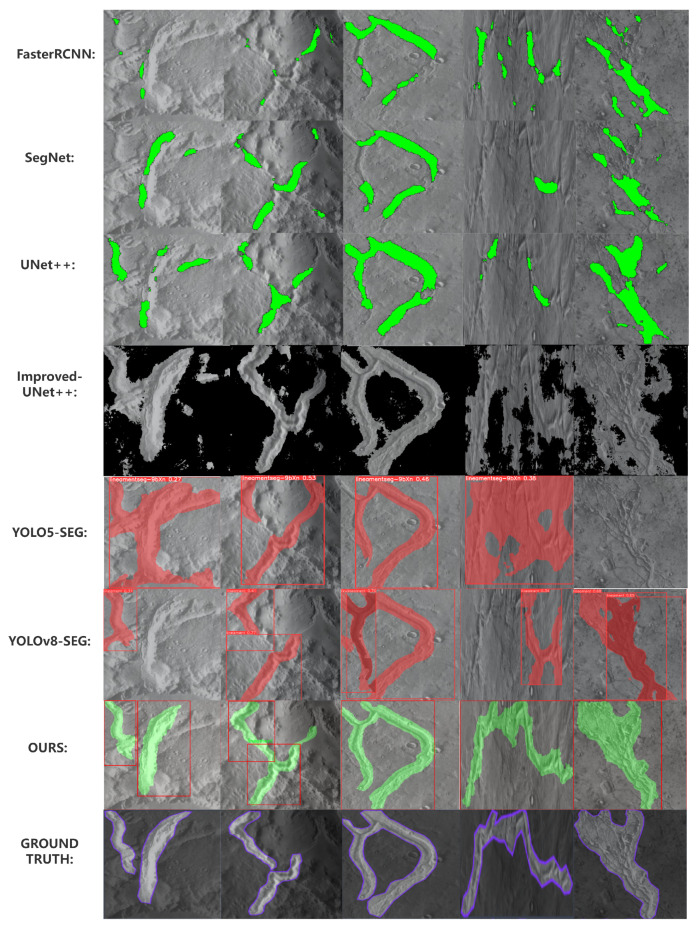
Comparison Experiment Results with Other Related Algorithms. It can be seen that less irrelevant pixels are detected in the result of the proposed method compared with other related algorithms. At same time it also shows clearer edge and bound box in the instance segmentation and object detection respectively of lineament structure result.

For the comparison of the proposed method and other related algorithms, we trained FCN [38], Faster-RCNN [39], SegNet [40], Deeplabv3 [41] YOLOv5-seg [42] and YOLOv8-seg [43] with the same strategy and evaluate metrics calculation method. The experiment results are shown in Table 2. It can be seen that the proposed method has relatively better and more stable performance compared with other related algorithms in the instance segmentation of lineament structure. It has the relatively higher value of mAP@50 and mAP@50:95. In most situation, if a model has a high mAP in any IoU threshold, it means that the model performs very well in object detection or semantic segmentation tasks, being able to accurately detect the presence of objects and having good detection performance across various categories of objects, shown in Figure 10.

**Figure 11:**
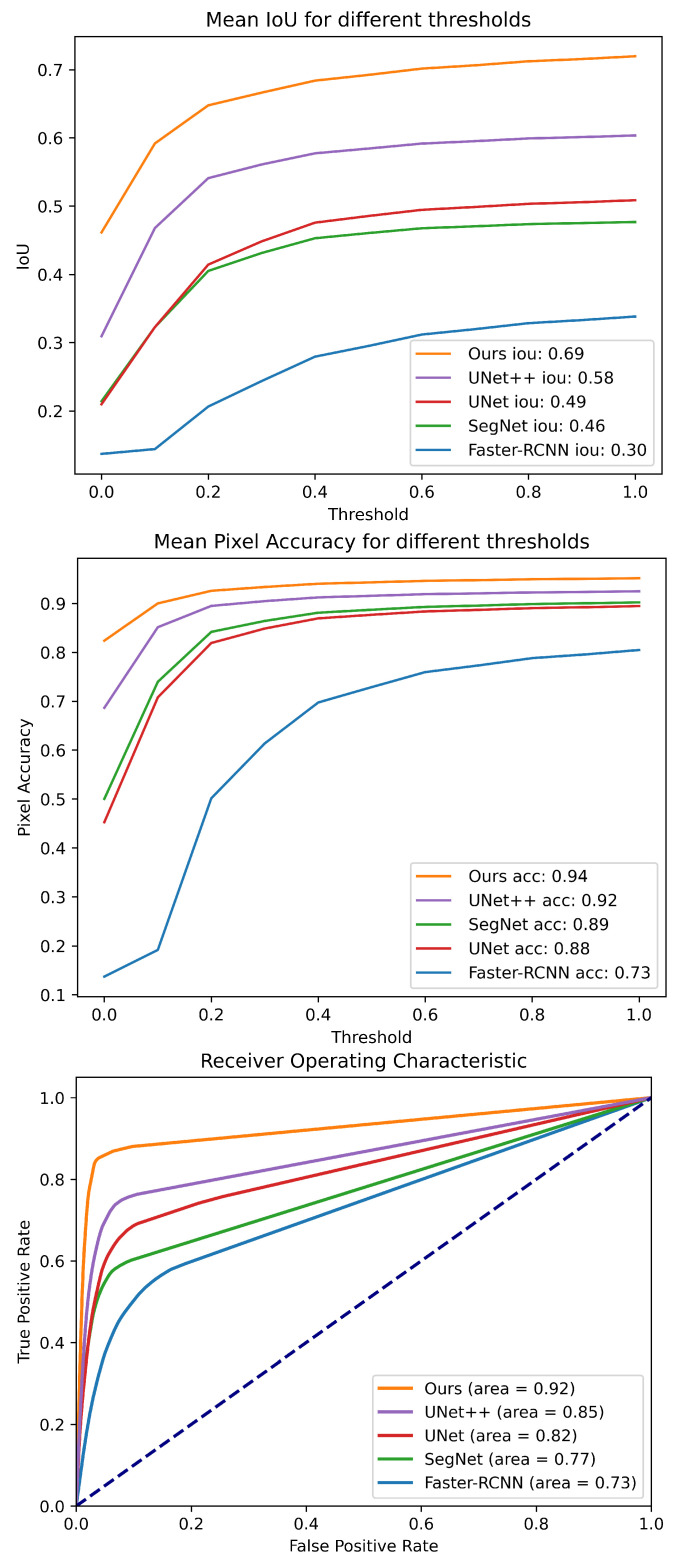
The Comparison of the Proposed Method and Other Related Algorithms in mean IoU and mean pixel accuracy under different IoU threshold and ROC curves. The IoU and mean pixel accuracy shown on the right are under threshold 0.5, which is the most common metric to evaluate the authenticity of samples. At same time, the ROC curves is calulated under the same threshold.

The experiment results show that our method have relatively better and more stable performance compared with other related algorithms in the instance segmentation of lineament structure. It can be seen that our method have the relatively higher value of mAP@50, mAP@50:95 mIoU and mean pixel accuracy in all tested thresholds accroding to Table 2 and Figure 11. At same time, it also shows that our ’polygon-match’ strategy algorithm is able to perform preciser edge detail in the instance segmentation of lineament structure result accroding to Figure 9.

## 5. Conclusions

A new Lunar lineaments structure landform extraction method is propoesed based on improved-UNet++ and YOLOv5 with polygon-match strategy algorithm. The method introduces a possible solution for the instance segmentation of Lunar lineament structure on the Lunar surface. First, a reliable Lunar lineament structure dataset is established based on the Lunar Orbital Data Explorer. Second, the improved-UNet++ and YOLOv5 networks are trained based on our new constructed dataset. Third, the polygon-match strategy algorithm is designed to improve the accuracy of the instance segmentation of lineament structure. Finally, the proposed method is compared with other related algorithms. The experiment results show that the proposed method has relatively better and more stable performance compared with other related algorithms in the instance segmentation of lineament structure. The proposed method has the relatively higher value of mAP@50 and mAP@50:95 compared with other related algorithms in the instance segmentation of lineament structure. The proposed method is able to extract Lunar lineament structure with preciser edge detail. It is able to provide a new solution for the instance segmentation of Lunar lineament structure on the Lunar surface. The proposed method has the potential to be applied in the geological exploration of Lunar surface, its accurate and efficient identification will contribute to the understanding of the geological history of the Moon and the exploration of the Moon’s resources and provide a reference for more accurate and detailed mapping of specific features of the Moon.

However, the proposed method still has some limitations. It contains following aspects. Firstly, we haven’t expended our dataset to other types of landform or lineament structure in other planet and test the performance of our improved-UNet++ and YOLOv5 networks. Due to the various and complexity geological activity in other planet, the lineament structure may has great difference compared with Lunar lineament. The generalization is still under verification. Secondly, an important prerequisite of our ’polygon match’ strategy algorithm is that the bounding boxes from YOLOv5 network should contain only correspond to one major lineament structure unit in most situation. However, in some situation, the bounding boxes may contain more than one lineament structure unit with similar size. The ’polygon match’ strategy algorithm is not able to perform well in this situation. The improvement of the ’polygon match’ strategy algorithm is still under consideration.

In the future, the data enhancement will be engaged in the future work. As the booming development of generative model [44], diffusion model [45], GAN model and other generative model will be applied in our future work [46], aiming to generate high quality and diversity lineament structure samples including building the lineament structure dataset from other planets. Furthermore, facing with restricted samples on some planets, for instance, Venus, where only few samples are recorded, we will try to generate more samples from the existing samples. At same time, we also plan to focus on establishing a standard to evaluate the result of generative results, which will guarantee the reality of the generated samples. Our final goal is to establish a lineament structure dataset with great quantity, high diversity and high quality. Meanwhile, we will also expend our dataset to other types of landform, aiming to improve the generalization ability of the proposed method. Simultaneously, we will also focus on the improvement of the network architecture, aiming to improve the performance of the proposed method, which aims to increase the ability of object detection and semantic segmentation.

## Figures and Tables

**Table 1:** Comparison Experiment Results of Improved-UNet++ and other UNet++ Networks.

Model	P	R	mAP@50	mAP@50:95
UNet++	0.4326	0.6452	0.5843	0.3493
Residual Block + UNet++	0.493	0.6642	0.6052	0.3786
CBAM + UNet++	0.40	0.39	0.0511	0.0979
Ours (Residual Block + CBAM + UNet++)	0.679	0.6766	0.6081	0.3887

**Table 2:** Comparison Experiment Results with Other Related Networks.

Model	P	R	mAP@50	mAP@50:95	Parameters	FLOPS
FCN	0.413	0.282	0.213	0.086	102,760,448	136.2 G
Faster-RCNN	0.481	0.344	0.296	0.103	19,234,523	239 G
SegNet	0.4377	0.451	0.3248	0.1422	29,461,472	165.2 G
Deeplabv3+	0.4526	0.4918	0.3943	0.1923	42,004,074	173.82 G
UNet++	0.4326	0.6452	0.5843	0.3493	9,207,472	56.88 G
YOLOv5-seg	0.623	0.477	0.464	0.147	7,398,422	25.7 G
YOLOv8-seg	0.623	0.477	0.464	0.147	3,258,259	12.0 G
Ours	0.679	0.6766	0.6081	0.3887	12,688,568	73.81 G

## Data Availability

Not applicable.
